# Supplementary material for: Osteoblastogenesis Alters Small RNA Profiles in EVs Derived from Bone Marrow Stem Cells (BMSCs) and Adipose Stem Cells (ASCs)
Source: Biomedicines. 2020 Sep 28;8(10):387. doi: 10.3390/biomedicines8100387 (PMC7599808; doi:10.3390/biomedicines8100387)
Supplement: Supplementary file 1 [file biomedicines-08-00387-s001.pdf]

Supplementary Table 1. The characteristics of cell donors.

Donor	Sex	Age	Ethnic group
BMSC 1	female	24	African American
BMSC 2	female	31	Non-Hispanic White
BMSC 3	female	18-25	NA
ASC 1	male	33	African American
ASC 2	female	44	African American
ASC 3	NA	NA	NA

NA for the ASC 3 because information cannot be sourced from the company anymore.

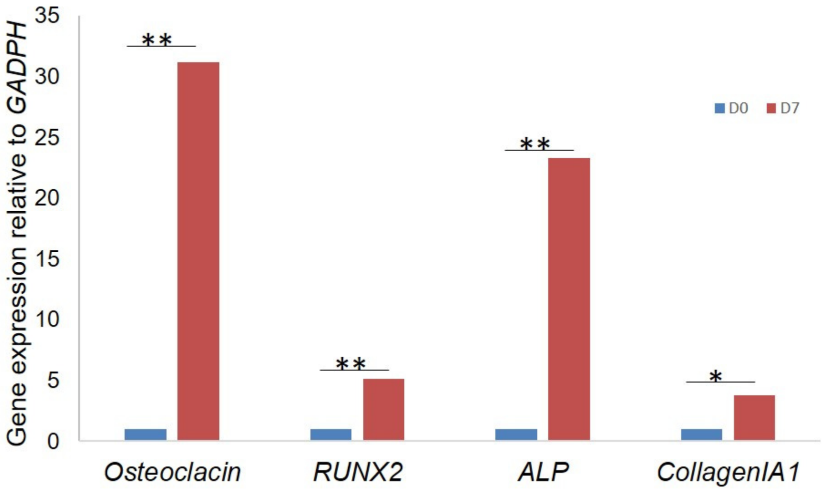

Supplementary Figure 1. QPCR quantification of gene expression of osteoblastic differentiation markers in BMSCs. D0 and D7 represent day zero and day seven of osteoblastic differentiation. GADPH was used to normalize the data. * p value<0.05; ** p value<0.005.
